# Potential Impact of Catch‐Up HPV Vaccination on HPV Prevalence and Cervical Cancer Incidence Among Women Living With HIV in South Africa: Results From Two Mathematical Models

**DOI:** 10.1002/jia2.70149

**Published:** 2026-07-24

**Authors:** Carla M. Doyle, Minttu M. Rönn, Cari van Schalkwyk, Marc Brisson, Nirali Soni, Marie‐Claude Boily, Mathieu Maheu‐Giroux

**Affiliations:** ^1^ Department of Epidemiology and Biostatistics, School of Population and Global Health McGill University Montréal Québec Canada; ^2^ Department of Global Health and Population Harvard T.H. Chan School of Public Health Boston Massachusetts USA; ^3^ The South African Centre for Epidemiological Modelling and Analysis Stellenbosch University Stellenbosch South Africa; ^4^ Centre de recherche du CHU de Québec Québec City Québec Canada; ^5^ Département de médecine sociale et préventive Université Laval Québec City Québec Canada; ^6^ Centre for Health Informatics, Computing, and Statistics Lancaster University Medical School Lancaster UK; ^7^ MRC Centre for Global Infectious Disease Analysis School of Public Health, Imperial College London London UK

**Keywords:** cervical cancer, HPV, HPV vaccination, mathematical modelling, women living with HIV

## Abstract

**Introduction:**

Women living with HIV (WLHIV) face an increased risk of cervical cancer (CC). With inequitable human papillomavirus (HPV) vaccine access globally and a programmatic focus on girls‐only school‐based delivery, many WLHIV in high HIV prevalence countries remain vulnerable to HPV and CC. We assessed the incremental impact of adding catch‐up vaccination for WLHIV in South Africa.

**Methods:**

We used two independently developed HPV/CC and HIV transmission models to predict the incremental impact of catch‐up HPV vaccination for WLHIV compared to routine‐only vaccination of girls aged 9−14 (90% cohort coverage) from 2020 onwards using a nonavalent vaccine (lifelong 100% protection). We assessed catch‐up scenarios vaccinating WLHIV aged 15−24 or ≥15 (attaining 90% cohort coverage), maintaining baseline CC screening. We report the predicted median annual prevalence of vaccine‐type high‐risk HPV (VT HR‐HPV), CC incidence and cumulative fraction of CC cases averted compared to routine‐only vaccination among WLHIV overall (age‐standardized) and by age.

**Results:**

With routine‐only vaccination, overall coverage among all WLHIV remained substantially (>50%) lower than in all women for 35−40 years, with gaps persisting even after 80 years. Adding catch‐up vaccination for WLHIV aged 15−24 increased coverage and benefits mainly among young WLHIV, with the largest annual reductions (relative to routine‐only vaccination) in VT HR‐HPV prevalence and CC incidence for WLHIV <30 years, reaching up to 36%–52% across models within 15 years and 38%–100% after 15−25 years, respectively. If catch‐up included WLHIV aged ≥15, overall vaccination coverage among WLHIV would immediately increase to 90%, extending benefits to older WLHIV—reducing peak annual relative reductions in CC incidence among WLHIV ≥50 by an extra 19% points compared to catch‐up vaccination of WLHIV aged 15−24, and shifting the peak 25 years earlier. Over 55−60 years, catch‐up vaccination of WLHIV aged 15−24 and ≥15 could avert up to 3%–8% and 14% of CC cases among WLHIV, respectively.

**Conclusions:**

Catch‐up vaccination of WLHIV can reduce their CC burden in the short to medium term, even in the presence of girls‐only routine vaccination programmes with high cohort coverage. To maximize impact, vaccines should be offered to WLHIV of all ages, not only younger women.

## 1 Introduction

Cervical cancer (CC) is preventable and curable when detected early. Yet, in 2022, approximately 660,000 women were diagnosed with, and 350,000 died from, CC globally [1]. The CC burden is disproportionately high in certain regions and populations, largely due to global inequities in prevention and treatment service access and the prevalence of risk factors, including HIV [2, 3, 4]. Eastern, southern, central and west Africa—home to 26 million people living with HIV (PLHIV) [5]—have the highest CC incidence and mortality [1, 3]. The World Health Organization (WHO) set programmatic CC elimination goals [6]; however, high HIV prevalence settings may require additional strategies.

Women living with HIV (WLHIV) face increased human papillomavirus (HPV) vulnerabilities, including heightened risks of acquiring and experiencing persistent high‐risk HPV (HR‐HPV) infection, and faster progression to cervical lesions and CC [7, 8]. While HIV antiretroviral therapy (ART) partially restores immune function, WLHIV on ART remain at elevated risk for these HPV‐related morbidities [7]. HPV vaccines are safe, produce a robust immunological response in PLHIV [9, 10], and can reduce WLHIV's CC risk and burden.

Over the past 20 years, three HPV vaccines became available: bivalent (HPV16/18), quadrivalent (HPV6/11/16/18) and nonavalent (HPV6/11/16/18/31/33/45/52/58) [11]. Initially licenced under three‐dose schedules, these vaccines showed near 100% seropositivity and efficacy against HPV‐related disease outcomes, with two‐ and, recently, one‐dose schedules showing non‐inferior immunogenicity and comparable protection against persistent HPV infection [11, 12, 13]. Despite these advancements, vaccine access across Africa remains suboptimal [14]. Rwanda launched Africa's first national HPV immunization programme in 2011 [14, 15]; by 2023, only 54% of countries had established programmes, and country‐level uptake varies widely (5%–99% first‐dose coverage) [14]. Most programmes offer school‐based delivery to cohorts of early adolescent girls [14, 16, 17], leaving many women, and particularly WLHIV, vulnerable to HPV and CC.

South Africa's HIV prevalence is among the highest, with 9% of adolescent girls and young women aged 15−24 years living with HIV (AGYW‐LHIV) in 2024 [18]. The country's public CC control strategy includes cytology‐based screening and, since 2014, school‐based two‐dose bivalent vaccination of girls generally aged 9−12 years [19]. National guidelines recommend screening 30‐ to 50‐year‐old women three times (every 10 years), or upon HIV diagnosis and every 3 years for WLHIV (or annually after a positive screen) [19]. A previous mathematical model comparison study assessed strategies for CC elimination by 2120 in South Africa [20]. While it found that vaccination and twice‐lifetime screening could achieve elimination in all women, it did not fully examine the added benefits of catch‐up vaccination for WLHIV.

We explored the incremental impact—beyond girls’ routine vaccination—of catch‐up vaccination for WLHIV in South Africa on HPV and CC outcomes using two mathematical models. We investigated how benefits accrue over time and across age groups, and considered variations in vaccination age, type, coverage, immunity duration and ART coverage (e.g. following global HIV funding cuts [21]).

## 2 Methods

### 2.1 Model Overviews

We leveraged two independently developed mathematical models of heterosexual HPV‐HIV co‐transmission in South Africa: *Det_HPV‐HIV* [20] and *MicroCOSM‐HPV* [20, 22, 23, 24]. Both simulate 13 HR‐HPV genotypes (vaccine types 16/18/31/33/45/52/58 and non‐vaccine types 35/39/51/56/59/68) and HIV/HPV interactions increasing persistent HPV and disease progression risks in WLHIV according to ART‐status (ART partially decreases persistent HPV risk and slows CC progression [25, 26])*. Det_HPV‐HIV* further assumes an increased HPV acquisition risk in PLHIV, and vice versa. CC screening reflects implementation in South Africa. HPV vaccination was initiated in 2014 (*MicroCOSM‐HPV*) or 2020 (*Det_HPV‐HIV*), with scenario‐specific coverage and eligibility (Table 1). Finally, both models were calibrated under Bayesian frameworks using demographic and epidemiologic data up to 2020.

**TABLE 1:** Modelled HPV vaccination scenarios from 2020 onwards in (A) main analysis and (B) sensitivity analysis.

			Routine vaccination (all)^a^		Catch‐up vaccination (WLHIV)^b^		
Scenario	Vaccine	Immunity duration	Age (years)	Cohort coverage	Age (years)	Cohort coverage	ART^c^
***(A) Main analysis***							
*Nonavalent versus bivalent vaccine with routine vaccination only*							
Comparator	Bivalent	Lifelong	9−14	90%	—	—	90‐90‐90
Scenario	Nonavalent	Lifelong	9−14	90%	—	—	90‐90‐90
*Catch‐up vaccination for WLHIV versus routine vaccination only (nonavalent vaccine)*							
Comparator	Nonavalent	Lifelong	9−14	90%	—	—	90‐90‐90
Scenario 1	Nonavalent	Lifelong	9−14	90%	15−24^d^	90%	90‐90‐90
Scenario 2	Nonavalent	Lifelong	9−14	90%	15−45^e^	90%	90‐90‐90
Scenario 3	Nonavalent	Lifelong	9−14	90%	≥15^e^	90%	90‐90‐90
***(B) Sensitivity analysis***							
*B.1. Vaccine type: Catch‐up vaccination for WLHIV versus routine vaccination only*							
Comparator	Bivalent	Lifelong	9−14	90%	—	—	90‐90‐90
Scenario	Bivalent	Lifelong	9−14	90%	15−24	90%	90‐90‐90
*B.2. Duration of vaccine immunity: Catch‐up vaccination for WLHIV versus routine vaccination only*							
Comparator	Nonavalent	20 or 30 years	9−14	90%	—	—	90‐90‐90
Scenario	Nonavalent	20 or 30 years	9−14	90%	15−24	90%	90‐90‐90
*B.3. Lower ART coverage: Catch‐up vaccination for WLHIV versus routine vaccination only*							
Comparator	Nonavalent	Lifelong	9−14	90%	—	—	2020 levels
Scenario	Nonavalent	Lifelong	9−14	90%	15−24	90%	2020 levels
*B.4. Lower catch‐up vaccination cohort coverage: Catch‐up vaccination for WLHIV versus routine vaccination only*							
Comparator	Nonavalent	Lifelong	9−14	90%	—	—	90‐90‐90
Scenario	Nonavalent	Lifelong	9−14	90%	15−24	50%	90‐90‐90
*B.5. Lower routine and catch‐up vaccination cohort coverage: Catch‐up vaccination for WLHIV versus routine vaccination only*							
Comparator	Nonavalent	Lifelong	9−14	80%	—	—	90‐90‐90
Scenario 1	Nonavalent	Lifelong	9−14	80%	15−24	50%	90‐90‐90
Scenario 2	Nonavalent	Lifelong	9−14	80%	15−24	80%	90‐90‐90

Abbreviations: ART, antiretroviral therapy; HPV, human papillomavirus; WLHIV, women living with HIV.

^a^ All scenarios in *Det_HPV‐HIV* assume no routine vaccination prior to 2020 for simplicity. In effect, cohorts eligible for vaccination between 2014 and 2020 are assumed to receive it in 2020, achieving equivalent coverage at the start of the scenario evaluation period.

^b^ Both models initiate catch‐up vaccination in 2020. *Det_HPV‐HIV* only modelled catch‐up vaccination of WLHIV aged 15−24, and its implementation lasted 3 years, after which the ageing‐in of routinely vaccinated girls was sufficient to maintain the target coverage in WLHIV aged 15−24. In *MicroCOSM‐HPV*, all catch‐up scenarios are modelled, with continuous implementation throughout the simulation period (although the number vaccinated diminishes with time, depending on age‐eligibility criteria). We do not expect services during the COVID‐19 pandemic to affect our results, as disruptions to routine HPV vaccination in South Africa were mainly limited to 2020, and the cohort of girls missed was included in the 2021 programme [24].

^c^ “*90‐90‐90*” indicates ART coverage scale up to meet the UNAIDS 90‐90‐90 targets by 2030 in all people living with HIV (men and women), reaching approximately 84% of all WLHIV. “*2020 levels*” indicates ART coverage plateaus in 2020, at approximately 70% of all WLHIV.

^d^ Referred to in the text as adolescent girls and young women living with HIV (AGYW‐LHIV).

^e^ Only *MicroCOSM‐HPV*.

### 2.2 Det_HPV‐HIV: Compartmental Model

*Det_HPV‐HIV* [20] is a dynamic compartmental model simulating three HR‐HPV groups (16/18, 31/33/45/52/58, and others not in the nonavalent vaccine) from 1950, with HIV introduced in 1985. The model is stratified by sex, 5‐year age groups (9−14 to 70−74 years) and sexual‐activity levels (low/medium/high). Condoms and male circumcision for HIV prevention are modelled, with time‐varying coverage per sex act. ART was introduced in 2004 (when South Africa's publicly funded programme launched), with coverage increasing towards 70% of PLHIV by 2020. Between 2000 and 2012, CC screening coverage in South Africa was low [1, 2, 27]. Thus, CC screening and treatment are modelled from 2012, with annual screening uptake plateauing in 2017 at 40% in 25‐year‐olds and 55% in 35‐ and 45‐year‐olds (without differences by HIV‐status) [28]. The model was calibrated to sex‐ and age‐specific data on national demographics, HIV prevalence, ART coverage, HR‐HPV, and cervical intraepithelial neoplasia grade 2 or worse (CIN2+) prevalence by HIV‐status, and CC incidence (Figure S1).

### 2.3 MicroCOSM‐HPV: Individual‐Based Model

*MicroCOSM‐HPV* [20, 22, 23, 24] is an individual‐based model simulating each HR‐HPV genotype and HIV from 1985 (beginning with 1 million individuals), considering sex, continuous age and sexual‐risk levels (low/high). Condom protection against HIV and HPV is considered, with coverage varying by time, sex, age and relationship type. ART is modelled from 2000, with uptake varying by time, sex and disease stage, reaching 70% coverage among PLHIV by 2020. CC screening and treatment began in 2000, with screening probabilities depending on age, time since last screen and ART‐status among WLHIV, estimated in calibration. Modelled screening coverage in 2019 was 45% among women not living with HIV aged 30−60 and 33% among WLHIV aged 25−60. The model was calibrated to national HIV prevalence, type‐specific HPV prevalence (by age and sex), CC screening coverages, CIN1 and CIN2/3 prevalence, and CC incidence (Figure S1).

### 2.4 Vaccination Scenarios

Each model simulated scenarios of girls‐only routine vaccination. Additional scenarios added on catch‐up vaccination for WLHIV in 2020 (Table 1), implemented to reach and maintain a target cohort coverage for 3 years in *Det_HPV‐HIV* and continuously in *MicroCOSM‐HPV*. Unless otherwise specified, scenarios attain 90% cohort coverages (i.e. 90% of age‐eligible girls or WLHIV vaccinated), use the nonavalent vaccine, assume 100% lifelong protection (without cross‐protection) and maintain 2019 CC screening assumptions. ART scale‐up reaches 81% coverage among PLHIV by 2030 (consistent with 90‐90‐90 targets), nearing 84% among WLHIV in both models (Figure S2). As HIV diagnosis is not modelled explicitly, catch‐up vaccination is applied to all WLHIV.

We first estimated the impact of using the nonavalent vaccine (vs. bivalent) from 2020 for routine‐only vaccination (no catch‐up) to understand the effect of the highest‐valency vaccine. We then assessed the incremental impact (vs. routine‐only vaccination) of adding catch‐up vaccination among AGYW‐LHIV and, using *MicroCOSM‐HPV*, WLHIV aged 15−45 or ≥15.

Sensitivity analyses assessed the incremental impact of catch‐up vaccination among AGYW‐LHIV using: (1) the bivalent vaccine; (2) shorter duration of vaccine‐induced immunity (20/30 years); (3) lower ART coverage (plateauing ∼70% of WLHIV in 2020; Figure S2); and (4) lower cohort coverages for routine and catch‐up vaccination (Table 1).

### 2.5 Outcomes

We predicted annual HPV vaccination coverage, annual VT and all HR‐HPV prevalence, and annual and cumulative new CC cases at baseline (2019) and over 2020−2100. We measured the additional population‐level impact of each catch‐up scenario compared to the relevant comparator (Table 1) by the annual relative reduction in HR‐HPV prevalence, annual relative reduction in CC incidence rates and cumulative fraction of CC cases averted (2020−2100). Outcomes were calculated among WLHIV overall (age‐standardized by 5‐year age groups to the South African UN Population Projections [29]) and by age.

### 2.6 Ethics

This study used simulated outputs from previously published mathematical models and did not require ethical approvals.

## 3 Results

### 3.1 Vaccination Coverage Across Scenarios

**FIGURE 1:**
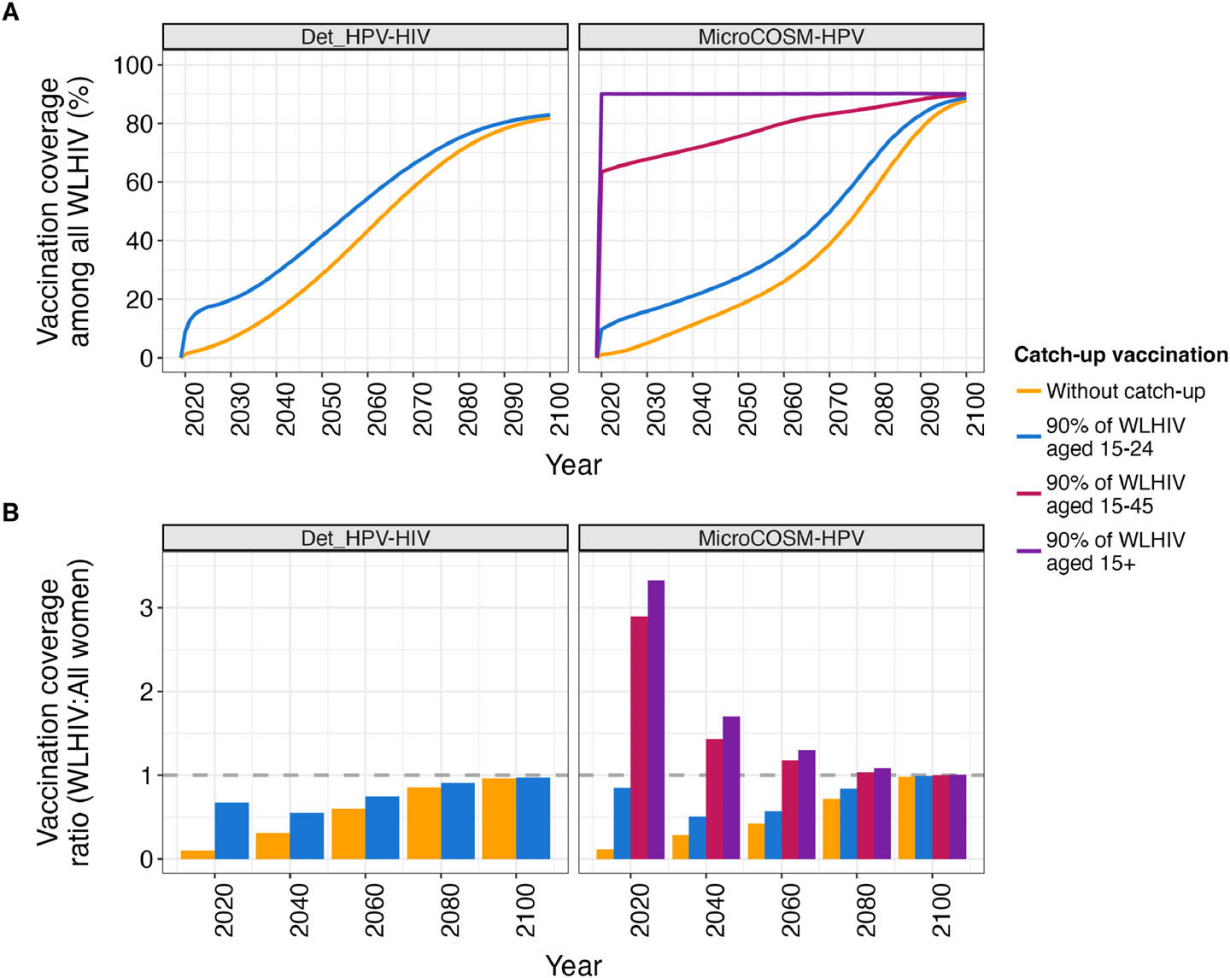
Overall vaccination coverage among women living with HIV (WLHIV). Panel A: The modelled overall vaccination coverage among all WLHIV over 2019−2100 under scenarios of routine vaccination in girls aged 9−14 years (90% cohort coverage) without and with catch‐up vaccination for WLHIV aged 15−24 (AGYW‐LHIV), WLHIV aged 15−45, and WLHIV aged ≥15 (90% cohort coverage). Panel B: The ratio of overall vaccination coverage between WLHIV and all women at 20‐year intervals between 2020 and 2100 under those same scenarios. Each panel presents the median estimates (across 27 and 100 posterior parameter sets in *Det_HPV‐HIV* and *MicroCOSM‐HPV*, respectively) per model. The colour indicates the scenario.

Following routine‐only vaccination, overall coverage among WLHIV steadily increased, as successive cohorts of girls were vaccinated (Figure 1A). However, given the increasingly older age profile of WLHIV due to declines in HIV incidence (Figure S3A,B), their vaccine coverage consistently lagged than of all women, remaining >50% below for 35−40 years (Figures 1B and S4). Adding catch‐up vaccination of AGYW‐LHIV increased overall coverage among WLHIV seven‐ to nine‐fold in 2020 and more than two‐fold until 2040 compared to routine‐only vaccination (with limited impact on coverage in all women), but the coverage gap between WLHIV and all women persisted for nearly 80 years (Figures 1B and S4). Eliminating this disparity required expanding catch‐up to WLHIV aged 15−45 or ≥15, which brought overall coverage among WLHIV to 63% and 90% in 2020, respectively, and increased coverage among all women two‐ to three‐fold compared to routine‐only vaccination (Figures 1A,B and S4). Furthermore, while coverage among AGYW‐LHIV rose rapidly across all catch‐up scenarios, without including older WLHIV (15−45 or ≥15 years), it took 65−75 years to reach high coverage (>70%) among WLHIV aged ≥50, who are most likely to develop CC (Figure S5).

### 3.2 Impact of Routine‐Only Vaccination

The median baseline (2019) VT HR‐HPV prevalence among WLHIV was 27% in both models (Figure S6A), with important age differences (Figure S8). Without vaccination, the prevalence among WLHIV modestly declined until 2030 (alongside ART scale‐up), and stabilized or continued to gradually decrease to 18%–23% over 2030−2100 (Figures S6A and S8). Routine‐only vaccination with the bivalent vaccine halved baseline VT HR‐HPV prevalence among WLHIV after approximately 60−75 years (by 2078−2093), and declined to 12%–13% across models in 2100 (Figure S7A), mainly driven by greater and more sustained decreases in younger women with high vaccination coverage (Figure S8). Routine‐only vaccination with the nonavalent vaccine halved the baseline prevalence among WLHIV about 40−50 years sooner than the bivalent vaccine (within 20 years, by 2038−2041), and surpassed a 90% decline after nearly 65 years (Figure S7A). Furthermore, all age groups experienced substantial prevalence declines with the nonavalent vaccine (Figure S8): <5% VT HR‐HPV prevalence within 11−36 years among <30‐year‐olds and 30−65 years among those aged ≥30, with overall prevalence <2% by 2092−2093 (Figure S7A). Figures S6B and S7B present all HR‐HPV types.

The median baseline CC incidence rate among WLHIV was 91−102 cases per 100,000 across models (Figure S5C), with higher rates in older WLHIV (108−264 per 100,000 in those aged ≥30 across models; Figure S9). Like HR‐HPV prevalence, without vaccination CC, incidence among WLHIV declined until 2030−2050 (Figure S6C), especially in those aged ≥40 (Figure S9). However, despite the long‐term HR‐HPV prevalence trends and subsequently stable age‐stratified CC incidence estimates, when age‐standardized, CC incidence in all WLHIV was predicted to increase to 104−107 cases per 100,000 by 2100 (Figure S6C). Routine‐only vaccination halved CC incidence among WLHIV after 52−72 years (by 2072−2092) and 48−52 years (by 2068−2072) with the bivalent and nonavalent vaccine, respectively, reaching 38−44 and 24−26 cases per 100,000 by 2100 (Figure S7C). All age groups experienced accelerated CC incidence declines under routine‐only vaccination—in the short‐term among WLHIV <40 and over time in WLHIV ≥40 —that were only slightly more pronounced with nonavalent vaccination (Figure S9).

Compared to all women, who showed similar long‐term HR‐HPV prevalence trends and stable CC incidence without vaccination (Figure S6A−C), CC rates were consistently higher for WLHIV—even after routine‐only vaccination (Figure S7A−C). However, baseline estimates among all women halved by routine‐only vaccination (bivalent or nonavalent) at comparable times as WLHIV. Routine‐only vaccination with the nonavalent vaccine had a greater impact on HR‐HPV prevalence than CC incidence, among both WLHIV and all women (Figure S10A−C). This translated into near‐negligible added CC benefits by using the nonavalent over the bivalent vaccine for routine‐only vaccination in the short‐ to mid‐term, averting <2% of cumulative CCs among WLHIV and all women within 28−55 years (Figure S11A,B). These added benefits increased gradually over time, but less substantially for WLHIV than all women—preventing 4%–14% and 6%–20% of CCs among WLHIV and all women, respectively, after 80 years (Figure S11A,B).

### 3.3 Impact of Adding Catch‐Up Vaccination for WLHIV on HPV Prevalence

**FIGURE 2:**
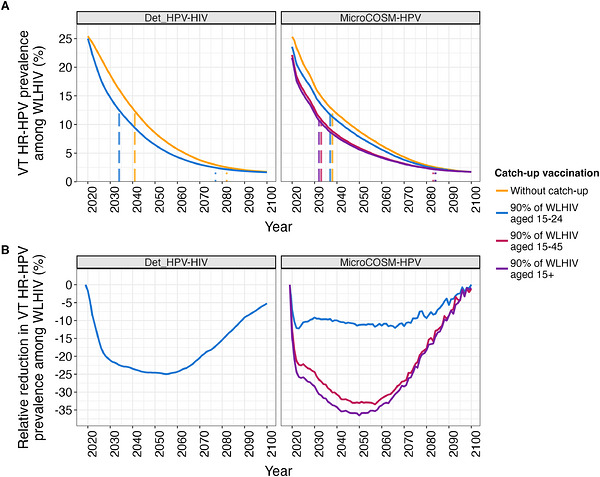
Impact of catch‐up vaccination on vaccine type (VT) high‐risk HPV (HR‐HPV) prevalence among women living with HIV (WLHIV). The annual estimated, age‐standardized VT HR‐HPV prevalence among WLHIV over 2019−2100 under scenarios of routine vaccination in girls aged 9−14 years (90% cohort coverage) without and with catch‐up vaccination for WLHIV aged 15−24 (AGYW‐LHIV), WLHIV aged 15−45, and WLHIV aged ≥15 (90% cohort coverage). Panel A: Absolute prevalence estimates. The vertical dashed and dotted lines indicate the year when VT HR‐HPV prevalence declined by 50% and 90% compared to the baseline (2019), respectively. Panel B: Relative reduction in prevalence of different catch‐up vaccination scenarios compared to routine‐only vaccination. Each panel presents the median estimates per model. The colour indicates the scenario.

Alongside increased vaccination coverage, adding catch‐up vaccination of AGYW‐LHIV and WLHIV ≥15 accelerated VT HR‐HPV prevalence declines among WLHIV—achieving 50% and 90% declines from baseline up to 6 years earlier than routine‐only vaccination across scenarios in both models (Figure 2A; Figure S12 shows all HR‐HPV types). Compared to routine‐only vaccination, catch‐up vaccination of AGYW‐LHIV or WLHIV ≥15 (*MicroCOSM‐HPV*) resulted in annual relative reductions in VT HR‐HPV prevalence among WLHIV by up to 12%–13% or 22%, respectively, within 5 years and 12%–25% or 37% within 40 years across models (Figure 2B).

**FIGURE 3:**
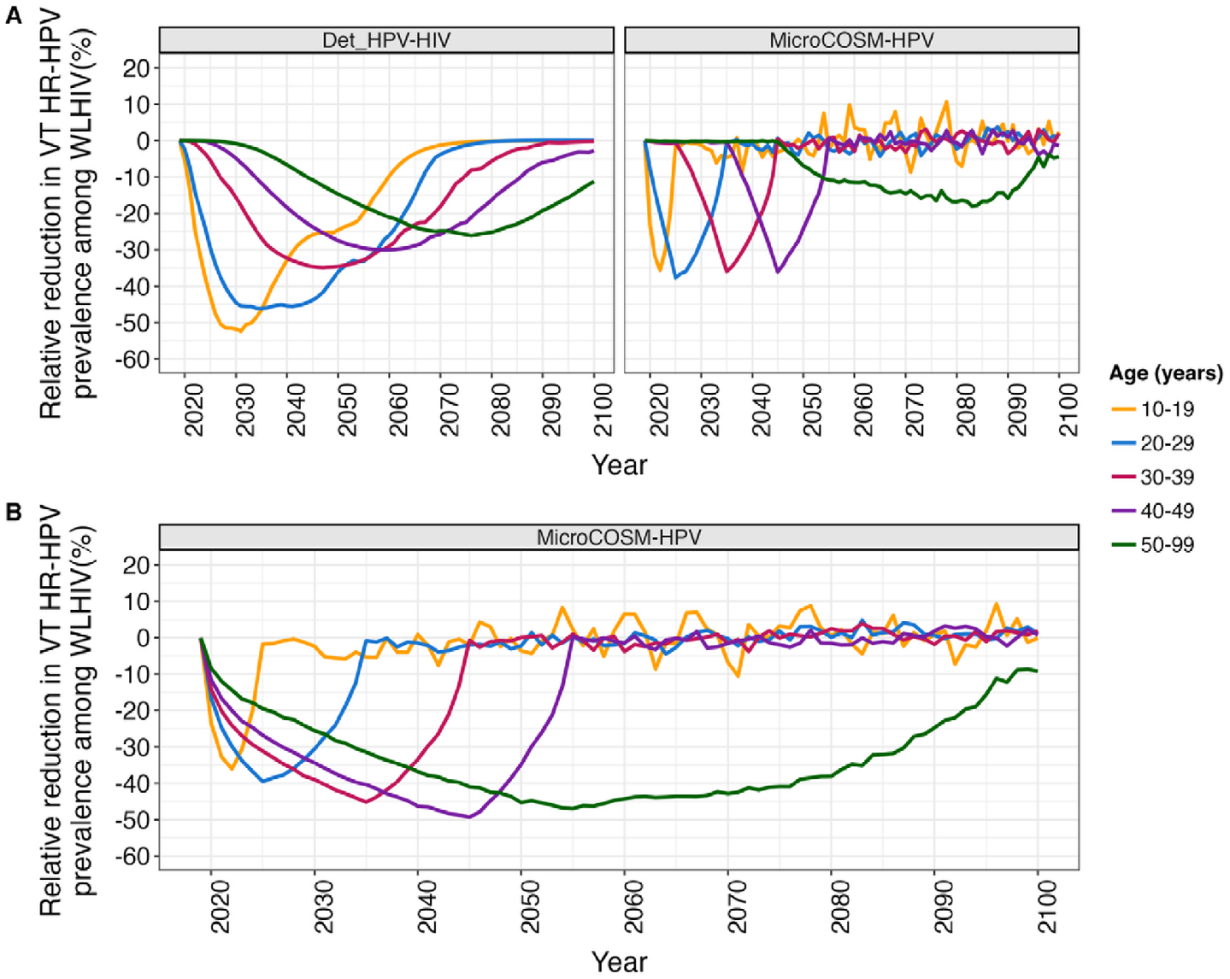
Age‐stratified relative reductions in vaccine type (VT) high‐risk HPV (HR‐HPV) prevalence among women living with HIV (WLHIV) compared to routine‐only vaccination. The relative reduction in annual estimated, age‐stratified VT HR‐HPV prevalence among WLHIV over 2019−2100 under scenarios of routine vaccination in girls aged 9−14 years (90% cohort coverage) with catch‐up vaccination for WLHIV aged 15−24 (AGYW‐LHIV; Panel A) and WLHIV aged ≥15 (Panel B) compared to routine‐only vaccination (90% cohort coverage). Each panel presents the median estimates per model. The colour indicates the age category.

Following catch‐up vaccination for AGYW‐LHIV, annual relative reductions (vs. routine‐only vaccination) in VT HR‐HPV prevalence among <30‐year‐old WLHIV reached 36%–52% across models within 15 years (2022−2036), but <36% across age groups of WLHIV ≥30 in both models after 15 years or more (Figure 3A). Expanding catch‐up to older WLHIV substantially benefited them (Figures 3B and S13–S16). Vaccinating WLHIV ≥15, the maximum annual relative reductions (vs. routine‐only vaccination) in VT HR‐HPV prevalence occurred at the same time (2035−2045 for 30−49 year‐olds) or 28 years sooner (2055 for ≥50‐year‐olds), and were further increased by 10%–13% points and 29% points in 30−49 and ≥50‐year‐olds, respectively, than when only vaccinating AGYW‐LHIV (Figures 3B and S13 and S14).

### 3.4 Impact of Adding Catch‐Up Vaccination for WLHIV on CC Incidence

**FIGURE 4:**
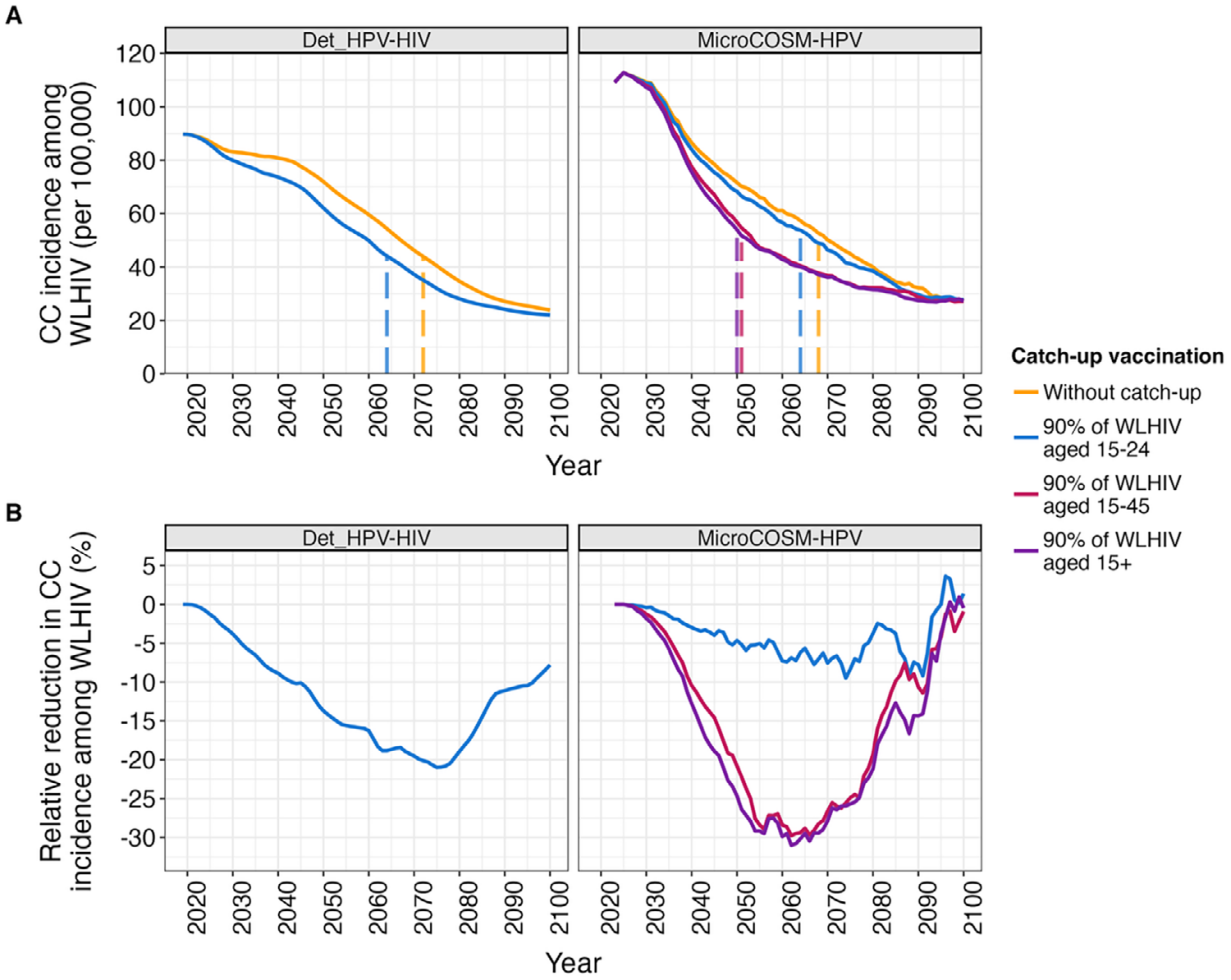
Impact of catch‐up vaccination on cervical cancer (CC) incidence among women living with HIV (WLHIV). The annual estimated, age‐standardized CC incidence among WLHIV over 2019−2100 under scenarios of routine vaccination in girls aged 9−14 years (90% cohort coverage) without and with catch‐up vaccination for WLHIV aged 15−24 (AGYW‐LHIV), WLHIV aged 15−45 and WLHIV aged ≥15 (90% cohort coverage). Panel A: Absolute CC incidence estimates. The vertical dashed lines indicate the year when CC incidence declined by 50% compared to the baseline (2019). Panel B: Relative reduction in CC incidence of different catch‐up vaccination scenarios compared to routine‐only vaccination. Each panel presents the median estimates per model. The colour indicates the scenario. A 5‐year simple moving average was applied to the *MicroCOSM‐HPV* estimates to smooth stochastic variations.

Catch‐up vaccination for AGYW‐LHIV and WLHIV ≥15 accelerated CC incidence declines, achieving a 50% from baseline 4−7 years (by 2064−2065 across models) and 18 years earlier (*MicroCOSM‐HPV*) than routine‐only vaccination, respectively (Figure 4A). Compared to routine‐only vaccination, catch‐up of AGYW‐LHIV began reducing annual CC incidence (relative reduction >5%) within 13−25 years across models (by 2033−2045), with reductions peaking at 14% and 21% after more than 55 years (2076−2091; Figure 4B). Expanding catch‐up to WLHIV ≥15 advanced the CC incidence impacts, with annual relative reductions (vs. routine‐only vaccination) >5% 12 years sooner than catch‐up of AGYW‐LHIV, and increasing to 35% within 30 years (by 2060; Figure 4B).

Following catch‐up of AGYW‐LHIV, annual relative reductions in CC incidence reached up to 38%–100% among <30‐year‐old WLHIV across models after approximately 15−25 years (between 2034 and 2053), while reaching a maximum of 21%–36% across age groups of WLHIV ≥30 after 25 years or more (2043–2091) across models (Figures S17 and S18). Like HR‐HPV prevalence, benefits to WLHIV ≥30 began earlier and were higher when catch‐up included older WLHIV (Figures S17 and S18).

### 3.5 Cumulative CC Averted by Adding Catch‐Up Vaccination for WLHIV

**FIGURE 5:**
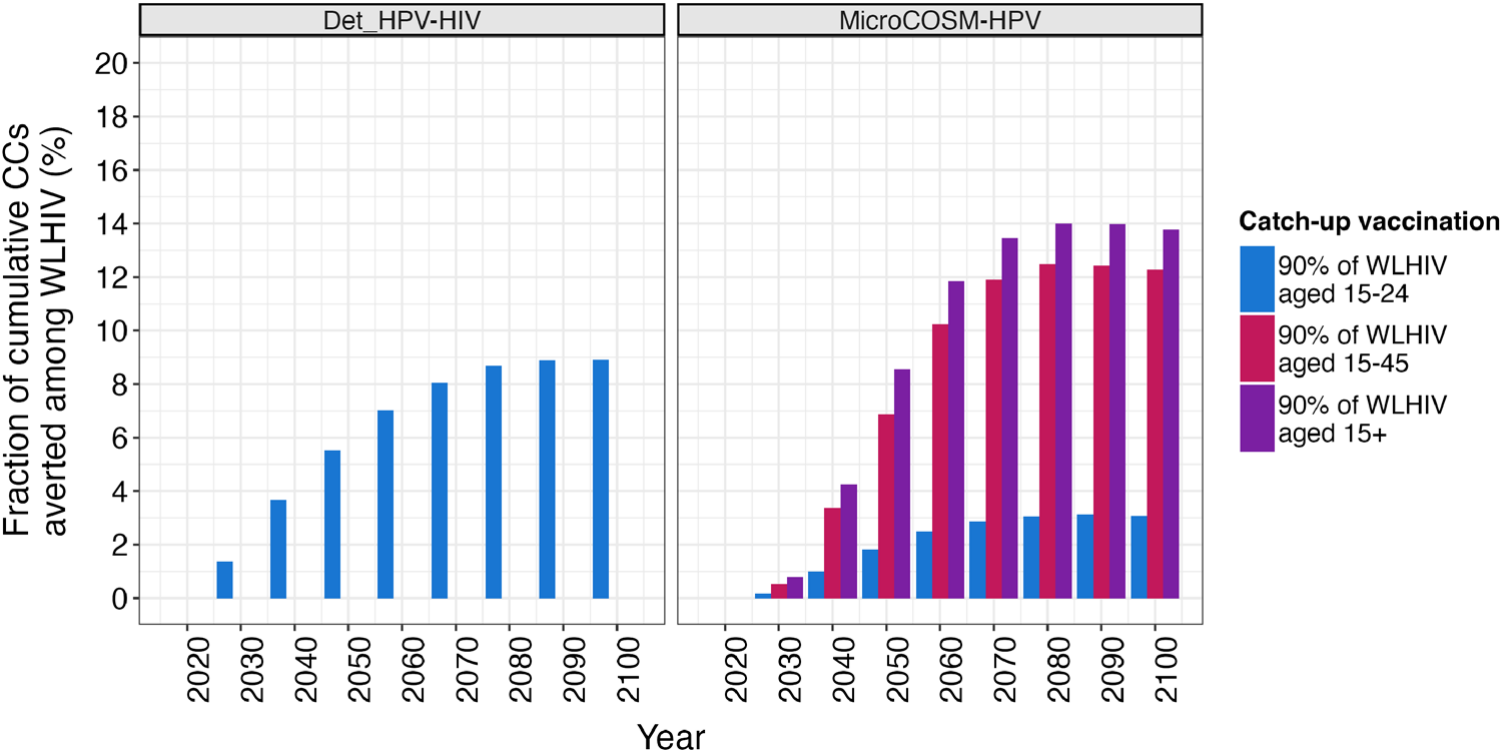
Incremental cumulative fraction of cervical cancer (CC) cases averted by catch‐up vaccination for women living with HIV (WLHIV) compared to routine‐only vaccination. The annual estimated, age‐standardized cumulative fraction of CC cases averted among WLHIV over 2020−2100 (at 10‐year intervals) under scenarios of routine vaccination in girls aged 9−14 years (90% cohort coverage) with catch‐up vaccination for WLHIV aged 15−24 (AGYW‐LHIV), WLHIV aged 15−45 and WLHIV aged ≥15 (90% cohort coverage) compared to routine‐only vaccination. Each panel presents the median estimates per model. The colour indicates the scenario.

Catch‐up vaccination of AGYW‐LHIV gradually averted more CC cases among WLHIV than routine‐only vaccination, taking 15−33 years to avert an extra 2% of cumulative CC cases (by 2035−2053) and 55 years to avert an extra 3%–8% across models (by 2074 in both), with little additional benefits thereafter (Figures 5 and S19 and S20). Expanding catch‐up to older WLHIV substantially accelerated and increased the additional benefits among WLHIV. Vaccinating WLHIV aged ≥15 averted >2% of cumulative CC cases 18 years sooner than catch‐up of AGYW‐LHIV (by 2035), accumulating benefits for approximately 60 years, averting an extra 14% of CC cases by 2080 (Figures 5 and S19).

**FIGURE 6:**
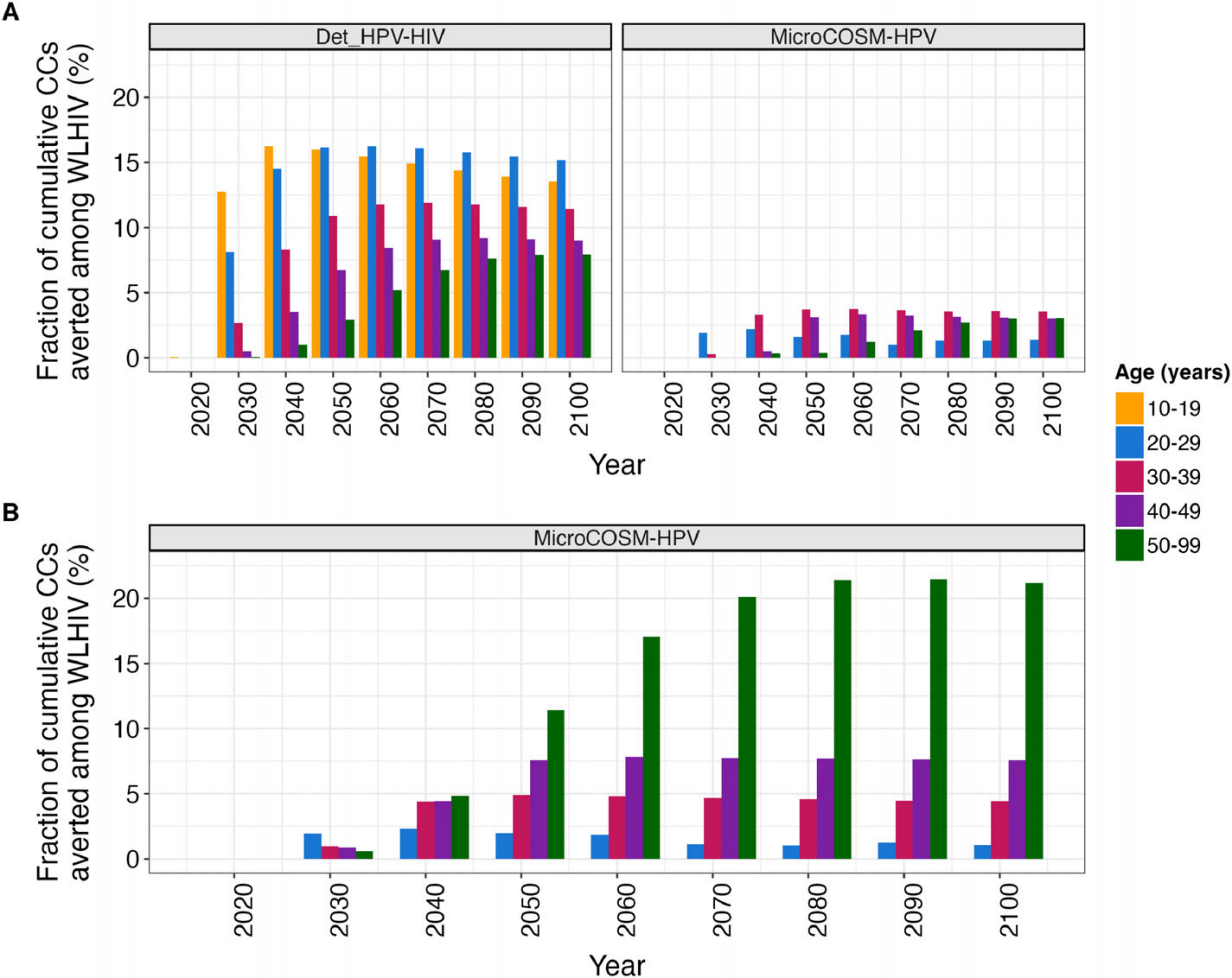
Age‐stratified incremental cumulative fraction of cervical cancer (CC) cases averted by catch‐up vaccination for women living with HIV (WLHIV) compared to routine‐only vaccination. The annual estimated, age‐stratified cumulative fraction of CC cases averted among WLHIV over 2020−2100 (at 10‐year intervals) under scenarios of routine vaccination in girls aged 9−14 years (90% cohort coverage) with catch‐up vaccination for WLHIV aged 15−24 (AGYW‐LHIV; Panel A) and WLHIV aged ≥15 (Panel B) (90% cohort coverage) compared to routine‐only vaccination. Each panel presents the median estimates per model. The colour indicates the age category. While both models incorporate a low CC incidence for young WLHIV, no CC cases occurred among WLHIV aged 10−19 years in *MicroCOSM‐HPV* in our set of stochastic simulations.

When vaccinating AGYW‐LHIV, there is a sequential shift in the timing of the maximum impact across age groups (due to ageing of vaccinated cohorts), with cohorts <30 years maximizing within 30−60 years (2030−2060), followed by progressively older cohorts until around 2080−2090 (Figures 6A and S21). Vaccinating WLHIV ≥15 accelerated impacts and averted a larger fraction of cumulative CC cases, especially among WLHIV aged 40−49 and ≥50 (Figures 6B and S21). In these age groups, approximately 8% and 21% of cumulative CC cases were averted by 2050 and 2080, respectively, when vaccinating WLHIV ≥15 (rather than 3% by 2051 and 4% by 2091, respectively, when vaccinating AGYW‐LHIV).

### 3.6 Sensitivity Analyses

**FIGURE 7:**
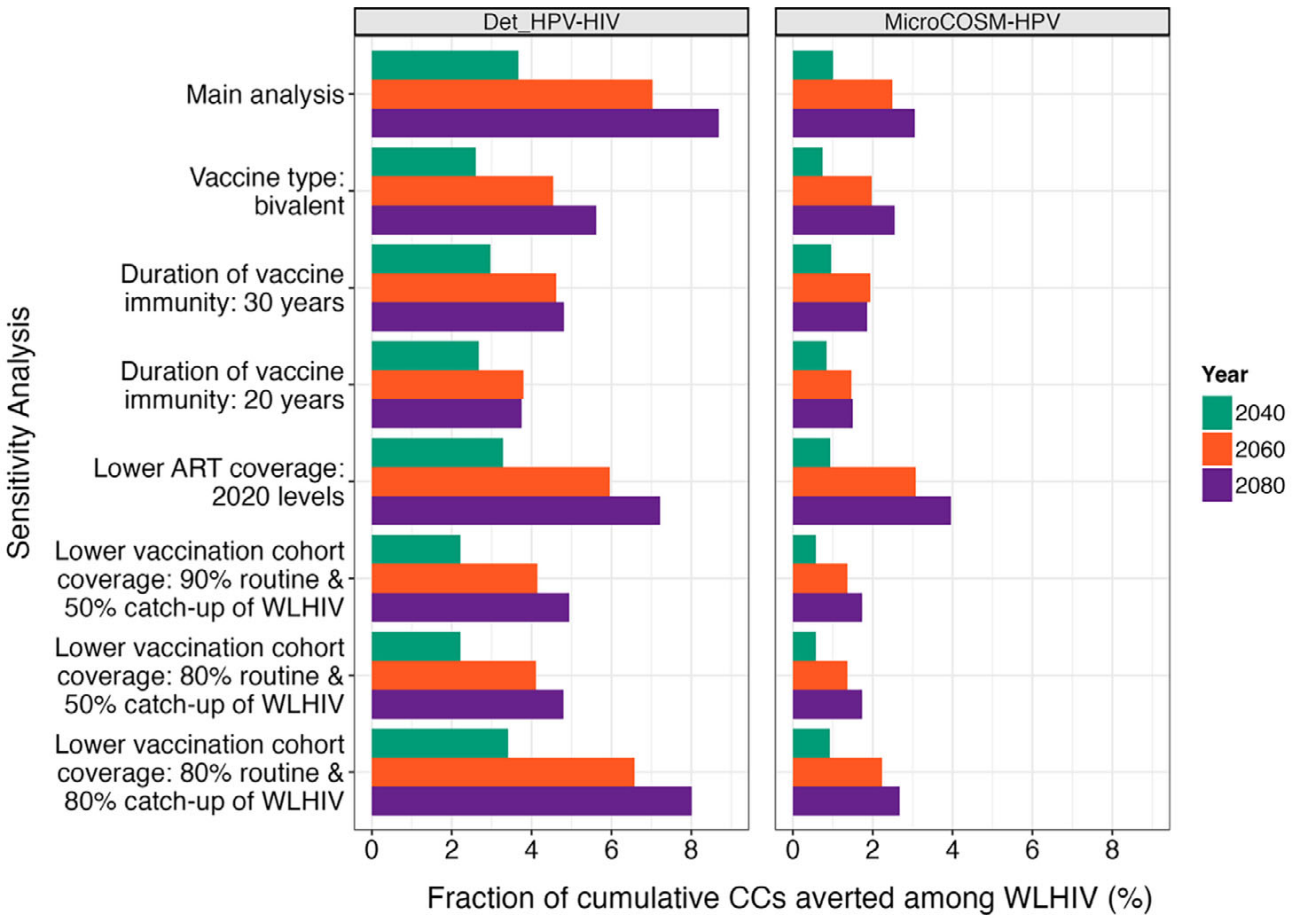
Sensitivity analysis of incremental impact of catch‐up vaccination of WLHIV (vs. routine‐only vaccination) on cumulative fraction of cervical cancer (CC) cases averted among WLHIV between 2020 and 2040, 2060, and 2080 under alternative conditions. The main analysis presented assumes catch‐up vaccination for WLHIV aged 15−24 years (AGYW‐LHIV) compared to routine‐only vaccination of girls aged 9−14 years (both with 90% cohort coverage) with the nonavalent vaccine, lifetime vaccine immunity and ART scaling up until 2030 to approximately 84%. Sensitivity analyses explored the same scenarios, but using the bivalent vaccine (lifelong immunity), shorter immunity duration (20 and 30 years; nonavalent vaccine), lower ART coverage (plateauing in 2020 near 70%), lower catch‐up cohort coverage (50%), and lower routine (80%) and catch‐up vaccination cohort coverage (50% and 80%) (details in Table 1B). Each panel presents the median estimates per model. The colour indicates the year.

The incremental impact of catch‐up vaccination for AGYW‐LHIV (90% cohort coverage) on CC cases (vs. routine‐only) would be reduced from 2040 if vaccine immunity lasts 20/30 years (instead of lifelong) or if using the bivalent vaccine (Figure 7). With 80% routine and catch‐up cohort coverage, results were similar to our main analysis; however, reducing catch‐up coverage to 50% attenuated the impact, being 45% lower than when cohort coverage is 90% by 2080 (Figure 7). Applying catch‐up to WLHIV on ART (*MicroCOSM‐HPV*) more gradually increased vaccine coverage among all WLHIV, delaying and attenuating CC impacts; by 2080, the cumulative fraction of CC cases averted by catch‐up for AGYW‐LHIV and WLHIV ≥15 (vs. routine‐only) was 30% and 22% lower, respectively, than when vaccinating all WLHIV (Figure S22). Finally, constant ART coverage from 2020 influenced the impact of catch‐up of AGYW‐LHIV (90% cohort coverage) differently across models (slightly lower in *Det_HPV‐HIV* and higher in *MicroCOSM‐HPV*), consistent with model differences in duration of catch‐up vaccination and HIV and ART interactions with HPV and CC (Figure 7).

## 4 Discussion

Routine HPV vaccination of young girls is fundamental for CC prevention. Our study used two dynamic transmission models to further investigate the impact of vaccinating WLHIV in South Africa. We found that adding catch‐up vaccination for WLHIV aged ≥15 could avert up to 14% more CC cases than routine‐only vaccination over 55−60 years. Restricting catch‐up to AGYW‐LHIV would avert a smaller fraction of CC cases (3%–8%). As WLHIV in South Africa are generally older than women not living with HIV, routine‐only vaccination leaves a critical vaccine coverage gap for up to 80 years—sustaining higher HPV prevalence and CC incidence for WLHIV. Catch‐up vaccination for AGYW‐LHIV would immediately boost coverage in those <30 years, before most acquire HPV, but could take up to 70 years to maximize its impact on CC incidence in WLHIV aged ≥30 years (relative to routine‐only vaccination). Instead, vaccinating WLHIV ≥15 would immediately close the coverage gap and reduce annual CC incidence, particularly among those aged ≥50, whose maximum reduction would occur 25 years sooner and be 19% points higher than when vaccinating AGYW‐LHIV.

Optimizing HPV vaccine allocation for WLHIV can inform strategies to reduce CC disparities. While ART scale‐up and routine CC screening could reduce persistent HR‐HPV infections and CC for WLHIV, like others [30], we showed they are insufficient on their own. Without any vaccination, our models predicted gradual HR‐HPV prevalence declines among WLHIV that leave levels high long‐term (18%–23%), and CC incidence could rise. South Africa currently uses the bivalent vaccine for routine vaccination, which our models suggest would slowly reduce VT HR‐HPV prevalence and CC incidence among WLHIV, taking up to 75 years for each to halve from baseline. Using the nonavalent vaccine for routine‐only vaccination would further reduce VT HR‐HPV and accelerate CC declines among WLHIV, each halving from baseline within 20 and 52 years, respectively. Despite the benefits of the nonavalent vaccine, the impacts of routine‐only vaccination are still more gradual than when catch‐up vaccination is implemented. Our qualitative conclusions about the additional benefits of catch‐up vaccination for WLHIV remain robust across our range of sensitivity analyses, providing high and long‐lasting vaccine‐induced immunity.

Despite consensus on its importance, few model‐based evaluations of CC control strategies jointly modelled HPV‐HIV [31, 32, 33, 34], a limited number of which examined the impacts for WLHIV [35, 36, 37, 38]. Those that did showed that, while routine vaccination can substantially reduce long‐term CC incidence for WLHIV, inequities persist, with CC rates remaining higher than in all women even with ART scale‐up [30, 39]. Our results align with a recent study based in KwaZulu‐Natal projecting that CC disparities between WLHIV and all women would only be reduced by targeted interventions for WLHIV [20, 30]. Recent *MicroCOSM‐HPV* analyses suggest catch‐up vaccination of WLHIV using the bivalent vaccine could be cost‐effective [24]. Nonavalent vaccination, however, was only evaluated for routine vaccination, and was not cost‐effective at current prices—though it may be at ∼USD 13−40 [24]. By extension, the cost‐effectiveness of nonavalent catch‐up vaccination among WLHIV likely depends on vaccine price, as well as dosing schedules.

The *WHO Africa Regional Immunization Technical Advisory Group* recommends prioritizing immunocompromised individuals [36]. Our findings provide timely insights for high HIV prevalence countries as HPV vaccination programmes scale up. Catch‐up vaccination for WLHIV would likely require a modest, short‐term investment in additional doses [24], potentially offset by shifts to one‐dose schedules for routine programmes, or otherwise supported by expanded manufacturing capacity and affordable pricing for GAVI‐supported countries [35, 36, 37]. However, our assumption of rapid scale‐up among WLHIV may be optimistic. Current recommendations of three doses for WLHIV, with known challenges in completing multi‐dose schedules, and suboptimal reach could slow implementation [40, 41]. Encouragingly, two recent South African evaluations of one‐ and two‐dose HPV vaccination in adolescent girls demonstrated substantial HPV prevalence reductions, with similar effectiveness by HIV‐status [42, 43]. Opportunities exist to integrate HPV vaccination with HIV services, with some clinics across Africa already doing so for AGYW‐LHIV eligible for the routine programme [16, 44]. Nonetheless, stakeholders highlight important operational challenges, including the complexity of delivering differentiated dosing schedules and ensuring confidentiality when prioritizing WLHIV [41]. Finally, the precarious funding environment raises concerns [21, 45]—uncertainty around GAVI support and constrained HIV funding could weaken service platforms needed to reach WLHIV and undermine ART coverage.

Our analyses have limitations. First, estimates are based on projections of the HIV and HPV epidemics in South Africa, which could be affected by future demographic and epidemiological trends. To minimize the potential impact of demographic shifts, overall estimates were age‐standardized to UN Population Projections for South Africa up to 2100 [20]. However, future intervention levels—particularly HPV or cervical screening—may influence results. Our projections are based on 2019 cervical screening coverage and frequencies, which, if increased, may overestimate the impact of catch‐up vaccination for WLHIV on CC incidence (i.e. fewer WLHIV may progress to CC if pre‐cancerous lesions are detected earlier). Second, both models have some structural limitations. For example, the deterministic model uses exponentially distributed ageing rates, resulting in some faster transitions through age compartments. The stochastic nature of the individual‐based model yielded highly variable age‐stratified CC incidence estimates, especially for younger ages. Third, uncertainties remain around the synergistic risks of HPV and HIV acquisition and disease progression for WLHIV. We address this uncertainty by presenting results from two independently developed models with different underlying assumptions and parameterizations, each calibrated under Bayesian frameworks. Finally, *Det_HPV‐HIV* began routine vaccination in 2020, although South Africa introduced bivalent vaccination in 2014, reaching 75% and 61% one‐ and two‐dose coverage, respectively, among 15‐year‐old girls in 2020 [46]. However, because routine vaccination in the model covers girls aged 9−14, most age‐cohorts eligible between 2014 and 2020 will simply be vaccinated in 2020 in our scenarios (i.e. equivalent coverage at the start of the model evaluation). Given the slow HPV prevalence decline for WLHIV under bivalent routine vaccination, this 6‐year delay should minimally affect our findings.

To our knowledge, this work is the first to isolate the impact of catch‐up vaccination for WLHIV, considering both HPV and CC outcomes among WLHIV overall and by age. Further, we offer an understanding of routine vaccination among all women and WLHIV using models that explicitly capture key HIV−HPV interactions and related interventions—an important limitation of prior evaluations [32]. By exploring a range of cohort coverages, age eligibility and vaccine characteristics, we comprehensively overview how programme design could maximize the impact of catch‐up vaccination for WLHIV [44].

## 5 Conclusions

Addressing the unique challenges faced by WLHIV in HPV prevention and control is essential for equitably achieving CC elimination goals. Catch‐up HPV vaccination for WLHIV can help reduce their CC burden, with benefits emerging earliest if offered to WLHIV of all ages. Prioritizing nonavalent vaccines and achieving high coverage in WLHIV are important for maximizing benefits.

## Author Contributions

CMD, MMR, CvS, MB, M‐CB and MM‐G contributed to the study conception and design. MMR, CvS and M‐CB contributed to model development, parameterization and calibration. Analyses of model outputs were performed by CMD, with support from MMR, CvS, M‐CB and MM‐G. The manuscript was drafted by CMD. All authors contributed to the interpretation of results and reviewed the manuscript for important intellectual content. Overall supervision for this project was provided by MM‐G and M‐CB. All authors approved the final manuscript.

## Funding

Grant from the *Canadian Institutes of Health Research* (CIHR) to MM‐G. MM‐G's research programme is funded by the *Tier 2 Canada Research Chair* in *Population Health Modelling*. M‐CB acknowledges funding from the *MRC Centre for Global Infectious Disease Analysis* (reference MR/R015600/1), jointly funded by the *UK Medical Research Council* (MRC) and the *UK Foreign, Commonwealth & Development Office* (FCDO), under the MRC/FCDO Concordat agreement and is also part of the EDCTP2 programme supported by the European Union. For the purpose of open access, M‐CB has applied a Creative Commons Attribution (CC BY) license to any Author Accepted Manuscript version arising. M‐CB, MMR and CvS acknowledge partial funding from the World Health Organization through the United States Agency for International Development (USAID) under the U.S. President's Emergency Plan for AIDS Relief (PEPFAR), and Unitaid.

## Conflicts of Interest

The authors declare no conflicts of interest.

## Prior Posting and Presentation

This work is the sole product of the authors and has never been submitted for publication. It has been presented at the *STI and HIV World Congress*. A pre‐print of this manuscript is available online at https://www.medrxiv.org/content/10.64898/2026.01.06.26343544v1.

## Supporting information




**Figure S1**: Select model calibration results.
**Figure S2**: Antiretroviral therapy (ART) coverage among women living with HIV.
**Figure S3**: Model age distribution of all women and women living with HIV (WLHIV) in the absence of routine or catch‐up vaccination.
**Figure S4** Vaccination coverage among women living with HIV (WLHIV) and all women under routine and catch‐up vaccination.
**Figure S5**: Vaccination coverage among women living with HIV (WLHIV) and all women by age under routine and catch‐up vaccination.
**Figure S6**: High‐risk HPV (HR‐HPV) prevalence and cervical cancer (CC) incidence among women living with HIV (WLHIV) and all women without vaccination.
**Figure S7**: High‐risk HPV (HR‐HPV) prevalence and cervical cancer (CC) incidence among women living with HIV (WLHIV) and all women under 90% routine‐only vaccination.
**Figure S8**: Impact of routine vaccination on vaccine type (VT) high‐risk HPV (HR‐HPV) prevalence among women living with HIV (WLHIV) and all women by age: absolute prevalence.
**Figure S9**: Impact of routine vaccination on cervical cancer (CC) incidence among women living with HIV (WLHIV) and all women by age: absolute incidence.
**Figure S10**: Impact of nonavalent vs. bivalent vaccination on high‐risk HPV (HR‐HPV) prevalence and cervical cancer (CC) incidence among women living with HIV (WLHIV) and all women under 90% routine‐only vaccination.
**Figure S11**: Cumulative fraction of cervical cancers (CC) averted by nonavalent vs. bivalent vaccination among women living with HIV (WLHIV) and all women under 90% routineonly vaccination.
**Figure S12**: Impact of catch‐up vaccination on high‐risk HPV (HR‐HPV) prevalence among women living with HIV (WLHIV) and all women.
**Figure S13**: Impact of catch‐up vaccination on vaccine type (VT) high‐risk HPV (HR‐HPV) prevalence among women living with HIV (WLHIV) and all women by age: absolute prevalence.
**Figure S14**: Impact of catch‐up vaccination on vaccine type (VT) high‐risk HPV (HR‐HPV) prevalence among women living with HIV (WLHIV) by age: relative reductions.
**Figure S15**: Impact of catch‐up vaccination on high‐risk HPV (HR‐HPV) prevalence among women living with HIV (WLHIV) and all women by age: absolute prevalence.
**Figure S16**: Impact of catch‐up vaccination on high‐risk HPV (HR‐HPV) prevalence among women living with HIV (WLHIV) by age: relative reductions.
**Figure S17**: Impact of catch‐up vaccination on cervical cancer (CC) incidence among women living with HIV (WLHIV) and all women by age: absolute incidence.
**Figure S18**: Impact of catch‐up vaccination on cervical cancer (CC) incidence among women living with HIV (WLHIV) by age: relative reductions.
**Figure S19**: Cumulative fraction of cervical cancers (CC) averted by catch‐up vaccination for women living with HIV (WLHIV) among WLHIV and all women.
**Figure S20**: Ten‐year difference in cumulative fraction of cervical cancers (CC) averted by catch‐up vaccination for women living with HIV (WLHIV) among WLHIV.
**Figure S21**: Age‐stratified cumulative fraction of cervical cancers (CC) averted by catch‐up vaccination for women living with HIV (WLHIV).
**Figure S22**: Impact of catch‐up vaccination for women living with HIV (WLHIV) when vaccinating all WLHIV or WLHIV on antiretroviral therapy (ART).

## Data Availability

Further information on the data is available upon request from MCB.
